# Reviewer acknowledgement 2013

**DOI:** 10.1186/1472-698X-14-1

**Published:** 2014-02-03

**Authors:** Irene Pala

**Affiliations:** 1 BioMed Central, 236 Gray's Inn Road, London, WC1X 8HB, UK

## Abstract

**Contributing reviewers:**

The editors of BMC International Health and Human Rights would like to thank all our reviewers who have contributed their time to the journal in Volume 13 (2013).

## 

Ramesh Adhikari

Nepal

Jones Adjei

Canada

Stephen Afranie

Ghana

Taha Ahmed

Sudan

Arif Alibhai

Canada

Gerardo Alvarez-Uria

India

Jimoh Amzat

Germany

Jehannine Austin

Canada

Kofi Awusabo-Asare

Ghana

Shamara Baidoobonso

Canada

Marian Bakermans-Kranenburg

Netherlands

Paul Bangirana

Uganda

Sarah Barber

South Africa

Sally Barlow

UK

Marian Barnes

UK

Corina Benjet

Mexico

Pauline Binder-Finnema

Sweden

James Blando

USA

Xavier Bosch-Capblanch

Switzerland

Paula Braitstein

USA

Garrett Brown

UK

Lisa Butler

USA

Gonca Celik

Turkey

Jeremiah Chakaya

Kenya

Venkatesan Chakrapani

India

Audrey Chapman

USA

Matthew Chersich

South Africa

Wong Chi Sang Martin

Hong Kong

Giuliana Cortese

Italy

Robert Cumming

Australia

Hannah Dahlen

Australia

Lucia D'Ambruoso

Sweden

Dena Davis

Uruguay

Manuela De Allegri

Germany

Ayesha De Costa

Sweden

Amare Deribew

Ethiopia

Ilse Derluyn

Belgium

Mai Do

USA

Michael Dunne

Australia

Gilles Dussault

Portugal

Matthew Ellis

UK

Kathryn Falb

USA

Seena Fazel

UK

Caryl Feldacker

USA

Jane Ferguson

Switzerland

Sharon Fonn

South Africa

Eric Friedman

USA

Owen Gallupe

Canada

Natalia Gavrilova

USA

Tesfay Gebregzabher Gebrehiwot

Ethiopia

Roger Gibson

Jamaica

Oliver Gruebner

Kenya

John Grundy

Cambodia

José Hagan

USA

Batool Haider

USA

Rachel Hammonds

Belgium

Heather Hartwell

UK

Lanis Hicks

USA

Jenni Hislop

UK

Eleanor Holroyd

Australia

Monjurul Hoque

South Africa

Sennen Hounton

USA

Allan House

UK

Hui-Chuan Hsu

Taiwan

Kun Huang

China

Junsheng Huo

China

Hyacinth Ichoku

Nigeria

Noelia Igareda

Spain

Leyla Ismayilova

USA

Michiyo Iwami

UK

Sara Javanparast

Australia

Ritsuko Kakuma

Australia

S M Mostafa Kamal

Bangladesh

Ali Karim

Ethiopia

Getnet Kassie

Ethiopia

Method Kazaura

Tanzania

Elissa Kennedy

Australia

Ines Keygnaert

Belgium

M Mahmud Khan

USA

Mobarak Hossain Khan

Germany

Emmanuel Koku

USA

Eugene Justine Kongnyuy

Cameroon

Paul Kowal

Switzerland

Marit Kramski

Australia

Caroline Kuo

USA

Vincent Z Kuuire

Canada

Yiannis Kyratsis

UK

Maria Caterina La Barbera

Spain

Amos Laar

Ghana

Mohammed Lamorde

Uganda

Heidi Larson

UK

Deb Levine

USA

Hagai Levine

Israel

Christos Lionis

Greece

Natasha Ludwig-Barron

USA

Timothy Mackey

USA

Kwok-Kei Mak

Hong Kong

James Manley

USA

Osman Mansoor

USA

Christine Markham

USA

Lawrence Mbuagbaw

Cameroon

Heather McCauley

USA

Jennifer McCleary-Sills

USA

William Miller

USA

Paul Mkandawire

Canada

Gillian Morantz

Canada

Malgorzata Mossakowska

Poland

Jin Mou

Canada

Bavon Mupenda

Democratic Republic of Congo

Maureen Murtaugh

USA

Richard Mutemwa

UK

Nicoli Nattrass

South Africa

Justin Needle

UK

David Newlands

UK

Nawi Ng

Sweden

Hazel Nichols

USA

Christiana Noestlinger

Belgium

Bright Nwaru

Finland

Constance Nyamukapa

Zimbabwe

Francis Obare

Kenya

Charles Oberg

USA

Okore Okorafor

South Africa

Hans Onya

South Africa

Joseph Oppong

USA

Maria José Osis

Brazil

David Osrin

UK

Adobea Owusu

Ghana

Gretel Pelto

USA

Amber Peterman

USA

Lancelot Pinto

India

Paola Pisani

Italy

Emma Plugge

UK

Tonia Poteat

USA

Gisela Priebe

Sweden

Erica Pufall

UK

Diane Putnick

USA

Zahidul Quayyum

UK

Anisur Rahman

Bangladesh

Motiur Rahman

Viet Nam

Davide Rasella

Brazil

Stephanie Raymond

France

Erica Richardson

UK

Linda Richter

South Africa

Michael Rowe

USA

Leonard Rubenstein

USA

Elizeus Rutebemberwa

Uganda

Shagun Sabarwal

India

Miguel San Sebastian

Sweden

Ayden Scheim

Canada

Federica Secci

UK

Sakthivel Selvaraj

India

Mikyong Shin

USA

Inga Dora Sigfusdottir

Iceland

Daudi Simba

Tanzania

Padam Simkhada

UK

Melissa Simon

USA

Irit Sinai

USA

Heather Sipsma

USA

Mary Smith Fawzi

USA

Robert Soeters

Netherlands

Egbert Sondorp

UK

Freddie Ssengooba

Uganda

Zachary Steel

Australia

Angelo Stefanini

Italy

Aslak Steinsbekk

Norway

Elizabeth Sully

USA

Elmuntasir Taha

Sudan

Augustinus ten Asbroek

UK

Eric Tenkorang

Canada

Brigit Toebes

Netherlands

Lil Tonmyr

Canada

Vivian Towe

USA

James K Tumwine

Uganda

Bart Van den Borne

Netherlands

Willem Herbert van Harten

Netherlands

Edwin van Teijlingen

UK

Ebrahim Variava

South Africa

Jennifer Wagman

USA

Abdul Wajid

USA

Joyce Wamoyi

Tanzania

Douglas Fraser Wares

Switzerland

Attiya Waris

Kenya

Lisa Welch

USA

Kathryn Whetten

USA

Daniel Fekadu Wolde-Giorgis

UK

Jonathan Wolff

UK

Kaspar Wyss

Switzerland

Eric Yeboah

Ghana

Hung-Wen Yeh

USA

Hatsumi Yoshii

Japan

Clement Zeh

Kenya

Eyob Zere

United Arab Emirates

George Zhang

USA

Shu Zhang

China

## Pre-publication history

The pre-publication history for this paper can be accessed here:

http://www.biomedcentral.com/1472-698X/14/1/prepub

